# Maternal plasma microRNA profiles in twin-twin transfusion syndrome and normal monochorionic twin pregnancies

**DOI:** 10.3389/fmolb.2025.1597215

**Published:** 2025-07-23

**Authors:** Steven T. Papastefan, Morgan M. Langereis, Catherine R. Redden, Daniel R. Liesman, Cassandra B. Huerta, Lucas E. Turner, Hee Kap Kang, Bethany T. Stetson, Katherine C. Ott, William S. Marriott, Joyceline A. S. Ito, Aimen F. Shaaban, Amir M. Alhajjat

**Affiliations:** ^1^ Department of Surgery, The Chicago Institute for Fetal Health, Ann and Robert H. Lurie Children’s Hospital of Chicago, Northwestern University Feinberg School of Medicine, Chicago, IL, United States; ^2^ Department of Surgery, Northwestern University Feinberg School of Medicine, Chicago, IL, United States; ^3^ Division of Maternal Fetal Medicine, Department of Obstetrics and Gynecology, Northwestern University Feinberg School of Medicine, Chicago, IL, United States

**Keywords:** microRNA, twin-twin transfusion syndrome, circulating biomarker, monochorionic diamniotic pregnancy, pregnancy

## Abstract

**Introduction:**

Ultrasound-based staging systems for twin-twin transfusion syndrome (TTTS) are limited by radiologic expertise, fetal positioning, and timing of the exam, and may benefit from incorporation of objective biochemical measures for diagnosis and prognostication. microRNA expression is altered in amniotic fluid of TTTS patients, however the invasive nature of amniocentesis has precluded practical incorporation of these biomarkers into current staging systems. Therefore, we sought to assess whether non-invasive maternal plasma microRNAs can distinguish between TTTS and normal monochorionic diamniotic (MCDA) twin pregnancies.

**Methods:**

Maternal blood samples were collected for patients with normal MCDA twin pregnancies (n = 11) or prior to selective fetoscopic laser photocoagulation (SFLP) for patients with TTTS (n = 36). Extracted microRNA from a panel of 24 microRNAs was compared between groups.

**Results:**

miR-26a-5p (P = 0.004), miR-222-3p (P = 0.007), and miR-145-5p (P = 0.047) were downregulated and miR-320a-3p (P = 0.005) was upregulated in the maternal plasma of TTTS patients compared to controls. miR-26a-5p, miR-320a-3p, and miR-222-3p in combination were strong predictors of TTTS on random forest modeling (area under curve = 0.905). After SFLP, all significantly dysregulated microRNAs in TTTS trended toward levels of expression observed in control MCDA twin pregnancies.

**Conclusion:**

Several microRNAs are differentially expressed in maternal plasma and demonstrate strong predictive capacity for identifying twin-twin transfusion syndrome. These plasma microRNAs could provide minimally invasive means to enhance currently established ultrasound diagnostic criteria for twin-twin transfusion syndrome.

## Introduction

Twin-twin transfusion syndrome (TTTS) complicates up to fifteen percent of monochorionic twin pregnancies and carries a high mortality rate when untreated (WAPM consensus group on Twin-to-twin transfusion syndrome et al., 2011). Imbalances in intertwin blood flow from placental arteriovenous anastomoses result in hypoperfusion of the donor twin and hyperperfusion of the recipient twin, with the latter manifesting polyhydramnios, cardiomegaly, and hydrops fetalis (Wohlmuth et al., 2016). Selective fetoscopic laser photocoagulation (SFLP) of the vascular anastomoses aims to modify the natural course of the disease prior to the onset of irreversible damage, however, early detection of twins that will benefit from SFLP remains a significant challenge (Manning and Archer, 2016; Behrendt and Galan, 2016; Crombleholme et al., 2007). Current staging systems of TTTS, including the Quintero, Cincinnati, and Children’s Hospital of Philadelphia systems, rely on fetal ultrasound and echocardiography (ECHO) measurements as the primary metrics signifying disease progression and informing the decision to proceed with SFLP (Quintero et al., 1999; Harkness and Crombleholme, 2005; Rychik et al., 2007). Shortcomings of the current staging systems include complete dependence on ultrasound which is operator dependent and can be affected by fetal positioning, maternal habitus or timing of exam. These factors lead to an inherit limitation to the staging system reflected in the heterogenous outcomes after SFLP (Stirnemann et al., 2010; Espinoza et al., 2021; Hessami et al., 2021; Rossi and D'Addario, 2009).

The incorporation of objective biochemical and genetic tests has revolutionized the diagnosis of fetal genetic and anatomic anomalies (Kim et al., 2023; Allyse et al., 2015) and may serve to complement existing ultrasound-based staging systems for TTTS by introducing operator and time independent mechanisms to evaluate TTTS. Prior studies have identified differences in the amniotic fluid biochemical milieu of patients with TTTS, however, the invasive nature of amniocentesis has precluded their use in clinical practice (Coleman et al., 2015; Dunn et al., 2016; Hoffman et al., 2020; Takano et al., 2001; Van Mieghem et al., 2010). Non-invasive biomarkers for TTTS, such as those obtained via maternal venipuncture, are comparatively rare, with prior studies only demonstrating differences in angiogenic biomarkers in maternal plasma of TTTS patients (Fox et al., 2010; Yinon et al., 2014). The paucity of identified maternal plasma biomarkers for TTTS stems conceivably from the wide range of maternal factors that influence plasma-level expression and differences in transplacental trafficking of certain types of biomolecules (Manokhina et al., 2017).

microRNAs (miRNAs) are short, 20–22 nucleotide non-coding ribonucleic acids (RNA) involved in posttranscriptional messenger RNA regulation. They have been demonstrated to transfer from the placenta to maternal plasma in both physiologic and pathophysiologic states, suggesting their potential role as non-invasive biomarkers for placental diseases such as TTTS (Luo et al., 2009; Higashijima et al., 2013). Though differences in miRNA expression exist within the amniotic fluid of TTTS patients (Schuchardt et al., 2022; Willner et al., 2021), no differences in miRNA expression were observed in maternal plasma in a recent, albeit underpowered, study (Mackie et al., 2019).

In this study, we aimed to compare the expression of a targeted panel of miRNAs in the maternal plasma of patients with TTTS and normal monochorionic diamniotic (MCDA) twin pregnancies. Given prior evidence for transplacental transfer of miRNAs and established differences present in amniotic fluid, we hypothesized that expression of maternal plasma miRNAs would differ between TTTS and normal MCDA twin pregnancies and could serve in a predictive capacity for the identification of TTTS via non-invasive sampling.

## Methods

### Study design

Patients with MCDA pregnancies referred to the Chicago Institute of Fetal Health at Ann and Robert H. Lurie Children’s Hospital between March 2020 and December 2023 were approached prospectively, and those consenting to collection of biological samples were enrolled. The Institutional Review Board of Ann and Robert H. Lurie Children’s Hospital approved this study (IRB #2020–3,250), and all subjects provided voluntary, written informed consent. All research was performed in accordance with relevant local guidelines/regulations as well as in accordance with the Declaration of Helsinki. TTTS was defined as monochorionic pregnancy with polyhydramnios in one sac (>8 cm maximum vertical pocket) and oligohydramnios in the other sac (<2 cm maximum vertical pocket) and no other apparent causes of amniotic fluid or growth discrepancy. Patients with a primary diagnosis of selective intrauterine growth restriction (sIUGR) with secondary evolution of TTTS were excluded due to inherent differences in miRNA expression that may be unrelated to TTTS (Kim et al., 2020). Furthermore, patients with twin anemia-polycythemia sequence, twin reversed arterial perfusion, or discordant fetal anomaly were excluded. Additional imaging parameters included recipient twin LV MPI, donor bladder visualization, absent or reversed UA end-diastolic flow, and reversed DV flow (Van Mieghem et al., 2009). Patients were staged according to Quintero and Cincinnati systems, and the presence of recipient twin cardiomyopathy was determined based on established Cincinnati criteria (Villa et al., 2014). For MCDA controls, the twin with the higher LV MPI was used for comparison. Additionally, GA at time of initial ultrasound/ECHO, SFLP, postoperative ultrasound/ECHO, and at delivery or fetal demise (if present) were collected.

### Sample collection

For TTTS pregnancies, blood was collected within 24 h prior to SFLP and amniotic fluid samples were collected at the time of SFLP. Additionally, post-procedure blood collection was performed for TTTS patients who returned for post-procedure follow-up within 5–7 days of SFLP. For uncomplicated MCDA pregnancies, blood was collected at the time of evaluation. Maternal blood was collected in ethylenediaminetetraacetic acid vacutainers (BD, Franklin Lakes, NJ), transferred on ice, and centrifuged at 1500 g for 10 min at 4°C. Amniotic fluid was collected in 60 mL syringes, transferred on ice, and centrifuged at 530 g for 10 min at 4°C. Supernatant from maternal plasma and amniotic fluid were aliquoted, snap frozen, and stored at −80°C.

### miRNA extraction and microarray

miRNA was purified from 200 µL of supernatants using miRNeasy kits and stored at −80°C prior to processing (Qiagen, Hilden, Germany). miRNA concentration and quality were assessed using the BioTek Epoch-2 Microplate Reader (Agilent, Santa Clara, CA). cDNA was synthesized from 30 ng miRNA with a custom Taqman 24-microRNA reverse transcriptase primer pool using the ProFlex polymerase chain reaction (PCR) system (Thermo Fisher, Waltham, MA). Pre-amplification of cDNA was performed using a custom Taqman microRNA pre-amplification primer pool and Fast Advanced Master Mix, and samples stored at −20°C. On day of analysis, pre-amplified cDNA products were thawed on ice and loaded into custom Taqman microarray cards in duplicate, and real-time PCR performed using Thermo Fisher QuantStudio 7 Flex Real-Time PCR System.

### Assembly of a custom 24 miRNA TTTS panel

A discovery panel of 380 miRNAs was assessed in amniotic fluid of seven patients with TTTS (four with cardiomyopathy, three without cardiomyopathy with stage I to III) undergoing SFLP to inform the composition of the custom miRNA panel. 114 miRNAs (30.0%) were expressed in total, and ten miRNAs were differentially expressed in TTTS with cardiomyopathy compared to without cardiomyopathy (Supplementary Figure S1A,B). A custom panel of 24 miRNAs was created using differentially expressed miRNAs from the discovery group. 15 additional miRNAs were considered to be biologically-plausible mediators of TTTS pathophysiology based on literature review, and U6 snRNA as an intended endogenous control (Supplementary Figure S1C) (Schuchardt et al., 2022; Willner et al., 2021; Mackie et al., 2019; Larrabee et al., 2005; Hromadnikova et al., 2015; Xu et al., 2021; Alvarado-Flores et al., 2022; Pelosi et al., 2020; Han et al., 2017).

### Statistical analysis

Descriptive statistics for clinical variables were performed using SPSS v30.0 (IBM, Armonk, NY). Continuous variables were compared via independent samples T-test for parametric data or Wilcoxon Rank-Sum test for nonparametric data. Categorical variables were compared via Fisher’s exact test.

Microarray data was exported from Expression Suite v1.3 (Thermo Fisher, Waltham, MA) to SPSS and R for statistical analysis. Duplicate cycle threshold (CT) values were averaged and miRNAs excluded from analysis if not expressed at CT ≤ 35 or in ≥75% of samples. Global miRNA normalization was performed as the intended control U6-snRNA was not present in any sample and other endogenous controls for this patient population are not established (Faraldi et al., 2019; Xiang et al., 2014). Delta-delta CT (ddCT) values were compared via Wilcoxon rank-sum test, and relative quantification (RQ) of miRNA expression between groups calculated via log_2_ transformations of ddCT values. Benjamini–Hochberg (B-H) false discovery rate (FDR) correction was performed, and both uncorrected and corrected P-values reported (threshold P < 0.05) (Benjamini and Hochberg, 1995). For comparisons of TTTS versus controls, a general linear model was used to control for gestational age (GA) at the time of sample collection.

Random forest regression was performed in R (www.R-project.org) to determine miRNAs with the highest predictive capacity for TTTS. The machine-learning model was used to generate and learn 500 potential decision trees, selecting the tree with the highest predictive capacity based on learning (n = 37) and test (n = 10) groups. Variable importance plots identified the top 3 predictive miRNAs for TTTS, and receiver operating characteristic (ROC) curves and hierarchical clustering trees were created for the combined top 3 miRNAs. Heat maps were generated in Expression Suite.

### Network and gene ontogeny pathway analysis

Putative targets of miRNAs were identified for the top 5 differentially expressed miRNAs (miR-26a-5p, miR-320-3p, miR-222-3p, miR-328-3p, miR-145-5p) using gene/miRNA enrichment via ClueGO/CluePedia plugins for Cytoscape (v3.10.1) (Ali et al., 2021; Bindea et al., 2013; Bindea et al., 2009). Gene targets of miRNAs were identified using miRanda (miRanda-miRNAs-v5-2012–07–19.txt.hz), and Kappa score ≥0.6 (miRanda score v5) used as a threshold for genes included in the network.

The genes identified via gene/miRNA enrichment were then subjected to gene ontogeny pathway enrichment analysis to analyze the relationships between miRNAs, target genes, and potential Gene Ontogeny (GO) pathways. A two-sided hypergeometric test was performed to identify pathways that were significantly enriched in the gene set (threshold B-H adjusted P < 0.05), and chord plots were created in SRplot (Tang et al., 2023).

## Results

### Patient characteristics

A total of 234 patients with MCDA pregnancies were evaluated during the study period, with 91 patients meeting inclusion criteria and 47 (51.6%) consenting for sample collection. Of these, 36 patients of these patients had TTTS and 11 patients were found to have uncomplicated MCDA twin pregnancies. Maternal and prenatal characteristics are displayed in Table 1 and individual patient characteristics in Supplementary Table S1. There was no significant difference in gestational age (GA) at initial ultrasound (20.5 ± 2.9 vs 20.1 ± 3.0, P = 0.789) or at sample collection between TTTS and controls (21.0 ± 2.7 vs 20.5 ± 3.3, P = 0.757). As expected, right ventricular (RV) and left ventricular (LV) myocardial performance index (MPI) were higher in TTTS compared to MCDA controls. Furthermore, umbilical artery (UA) and ductus venosus (DV) waveform abnormalities and non-visualization of the donor twin bladder were observed only in TTTS patients. There was a trend toward significance for overall fetal survival (70.8% vs 90.9%, P = 0.087) between TTTS patients and controls (for whom SFLP was not indicated). The sole fatality in the control group had a normal ultrasound but developed preterm premature rupture of membranes of unknown etiology at 17^+6^ weeks.

**TABLE 1:** Clinical characteristics of the cohort.

Characteristic	Control (n = 11)	TTTS (n = 36)	P-value^a^
Maternal age at initial ultrasound (years)	32.8 ± 6.5	31.1 ± 5.3	0.576
GA at initial ultrasound (weeks)	20.1 ± 3.0	20.5 ± 2.9	0.789
GA at blood sample collection (weeks)	20.5 ± 3.3	21.0 ± 2.7	0.757
GA at SFLP (weeks)	-	21.1 ± 2.8	-
GA at delivery (weeks)	33.2 ± 5.9	30.8 ± 4.5	0.018
Recipient Twin LV MPI^b^	0.36 ± 0.06	0.48 ± 0.11	<0.001
Recipient Twin RV MPI^c^	0.32 ± 0.07	0.53 ± 0.22	<0.001
Recipient Twin Cardiomyopathy^d^	0	28 (55.6%)	<0.001
UA or DV waveform abnormality	0	18 (50.0%)	0.003
Absent or small donor bladder	0	28 (78.8%)	<0.001
Quintero Stage			
Stage 1	-	9 (25.0%)	-
Stage 2	-	7 (19.4%)	-
Stage 3	-	20 (55.6%)	-
Cincinnati Stage^e^			
Stage 3A	-	7 (19.4%)	-
Stage 3B	-	10 (27.8%)	-
Stage 3C	-	11 (30.6%)	-
Demise of Both Twins (of total pregnancies)	1/11 (9.1%)	6/36 (16.7%)	1.000
Demise of One Twin (of total pregnancies)	0	9/36 (25.0%)	0.092
Overall Fetal Survival (of total twins)	20/22 (90.9%)	51/72 (70.8%)	0.087

^a^ Wilcoxon rank-sum test for continuous variables, Fisher’s exact test for categorical variables.

^b^ Available for 33 of 36 TTTS, pregnancies and 11 of 11 controls.

^c^ Available for 32 of 36 TTTS, pregnancies and 11 of 11 controls.

^d^ Available for 32 of 36 TTTS, pregnancies.

^e^ For patients with recipient twin cardiomyopathy.

Data presented as mean ± SD, or n (%).

### Maternal plasma miRNAs are differentially expressed in TTTS compared to MCDA controls

**FIGURE 1:**
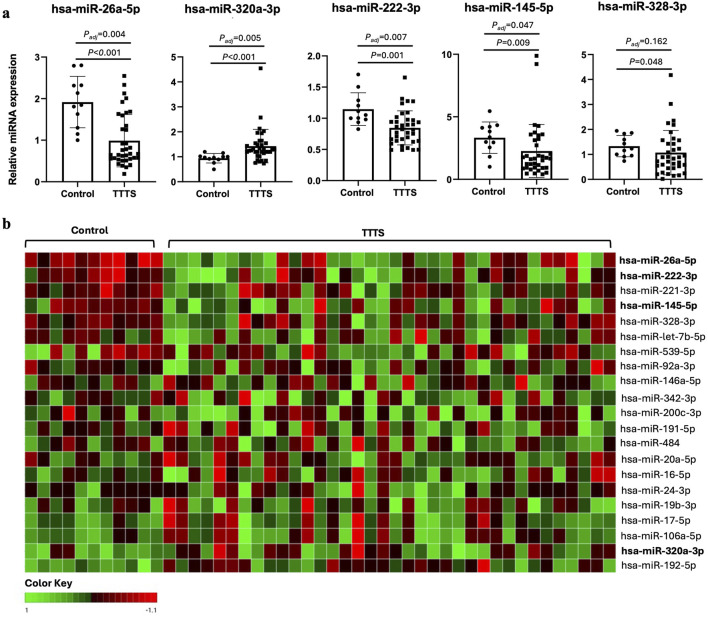
Differential expression of miRNAs in controls versus TTTS. **(a)** Bar graphs depicting relative miRNA expression of the top 5 differentially expressed miRNAs between controls (n = 11) and TTTS (n = 36). Individual patients represented as dots, and bars represent mean ± standard deviation. Asterisk denotes Benjamini–Hochberg adjusted P-value <0.05. **(b)** Heat map reflecting relative ddCT of respective miRNA in controls versus TTTS patients. Green boxes represent higher ddCT and red boxes represents lower ddCT, and therefore lower and higher relative expression compared to the globally normalized reference, respectively.

**FIGURE 2:**
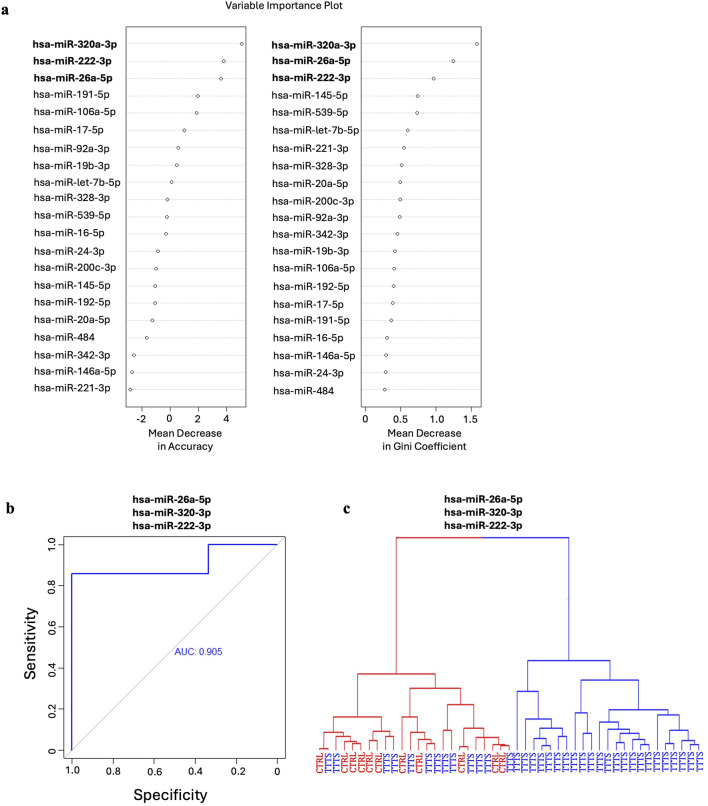
Random forest analysis of the predictive capacity of miRNAs for TTTS. **(a)** Mean decrease in accuracy and mean decrease in Gini coefficients reflect the respective predictive capacity of each miRNA for identifying TTTS. The top three miRNAs in both models, hsa-miR-320a-3p, hsa-miR-26a-5p and hsa-miR-222-3p were utilized in the random forest model for their combined predictive capacity for TTTS. **(b)** ROC curve for the top three miRNAs in the random forest model depicting strong predictive capacity for TTTS as indicated by an area under the curve (AUC) of 0.905. **(c)** Hierarchical clustering of patients using the model demonstrates two clusters composed entirely of patients with TTTS, and one cluster containing both TTTS and control patients.

After FDR correction, miR-26a-5p, miR-222-3p, and miR-145-5p were significantly downregulated and miR-320a-3p was significantly upregulated in TTTS maternal plasma compared to controls (Figure 1a). Though miR-328-3p was significantly upregulated in TTTS patients, this difference did not persist after FDR correction. Similarly, after controlling for GA at collection, the differences in miR-26a (P < 0.001), miR-320a (P = 0.015), miR-222 (P = 0.006), and miR-145 (P = 0.035) remained significant. Comprehensive comparison of analyzed miRNAs between TTTS and controls is displayed in Table 2 and depicted in the heat map (Figure 1b). On random forest prediction modeling, miRNAs were ranked according to their variable importance in predicting the occurrence of TTTS (Figure 2a). The predictive capacity of the top three discriminating miRNAs in the model (miR-26a-5p, miR-320a-3p and miR-222-3p) is demonstrated by the ROC curve (Figure 2b). Hierarchical clustering demonstrated segregation of 23 of 36 TTTS patients into a discrete cluster and 13 TTTS patients clustering with controls (Figure 2c).

**TABLE 2:** Differential expression of miRNAs between patients with TTTS and control MCDA twin pregnancies.

Assay	Mean ∆CT TTTS (SD)	Mean ∆CT control (SD)	∆∆CT (TTTS – Control)	Fold change (TTTS/Control)	P-value	B-H Adj. P-value
hsa-miR-26a-5p	4.63 (0.89)	3.48 (0.50)	1.15	0.52	<0.001	0.004
hsa-miR-320a-3p	−0.15 (0.52)	0.38 (0.34)	−0.53	1.51	<0.001	0.005
hsa-miR-222-3p	−0.71 (0.45)	−1.19 (0.31)	0.48	0.74	0.001	0.007
hsa-miR-145-5p	4.60 (1.13)	3.72 (0.69)	0.88	0.68	0.009	0.047
hsa-miR-328-3p	−1.08 (1.50)	−1.89 (0.48)	0.80	0.80	0.048	0.162
hsa-miR-192-5p	6.50 (1.15)	7.15 (0.66)	−0.65	2.12	0.051	0.162
hsa-miR-221-3p	−0.26 (1.14)	−0.94 (0.49)	0.68	0.73	0.054	0.162
hsa-miR-let-7b-5p	−0.55 (0.89)	−0.97 (0.46)	0.42	0.86	0.081	0.213
hsa-miR-17-5p	−1.61 (0.76)	−1.24 (0.29)	−0.37	1.47	0.190	0.443
hsa-miR-92a-5p	−4.96 (1.65)	−5.60 (0.91)	0.64	1.02	0.227	0.451
hsa-miR-106a-5p	−1.26 (0.68)	−0.95 (0.21)	−0.32	1.38	0.236	0.451
hsa-miR-342-3p	−0.01 (0.84)	−0.17 (0.46)	0.16	1.03	0.314	0.550
hsa-miR-200c-3p	7.59 (1.21)	7.05 (1.59)	0.54	0.37	0.415	0.651
hsa-miR-539-5p	5.85 (1.18)	5.65 (1.59)	0.20	0.78	0.434	0.651
hsa-miR-191-5p	1.04 (0.76)	1.03 (0.22)	0.02	1.13	0.594	0.832
hsa-miR-20a-5p	2.12 (0.78)	2.07 (0.61)	0.05	1.04	0.665	0.873
hsa-miR-19b-3p	0.06 (0.76)	0.16 (0.31)	−0.10	1.25	0.738	0.883
hsa-miR-484	−3.61 (0.06)	−3.54 (0.38)	−0.08	1.11	0.757	0.883
hsa-miR-146a-5p	0.07 (0.64)	0.05 (0.40)	0.02	1.05	0.951	0.990
hsa-miR-24-3p	0.78 (0.67)	0.87 (0.37)	−0.09	1.16	0.951	0.990
hsa-miR-16-5p	−1.99 (1.01)	−1.92 (0.40)	−0.07	1.30	0.990	0.990

### SFLP for TTTS returns miRNA expression to the direction of control MCDA pregnancies

**FIGURE 3:**
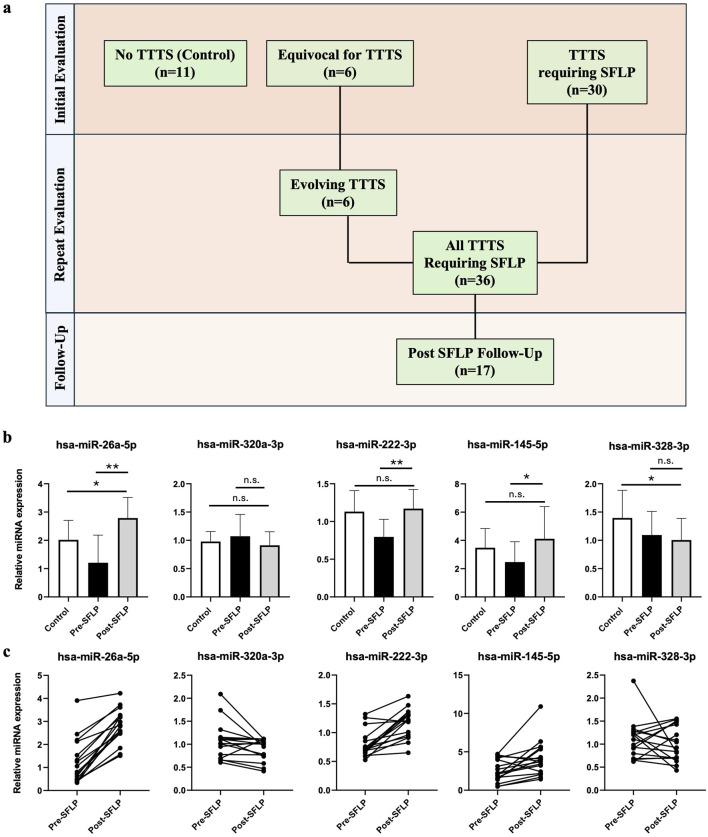
Comparison of miRNA expression at multiple time points with respect to SFLP response. **(a)** Characteristics of the patient cohort. At initial presentation, patients referred to the fetal treatment center were either confirmed to have TTTS requiring SFLP (n = 30) or had an MCDA pregnancy without TTTS or with TTTS wherein SFLP was not indicated (n = 17). All patients in the latter group returned for repeat examination and were found to either have evolving TTTS (n = 6) or no further evidence of TTTS (n = 11). A total of 36 patients with TTTS underwent SFLP, and 17 patients underwent maternal blood sample collection on post-SFLP follow-up. **(b)** Relative miRNA expression of the top 5 differentially expressed miRNAs before and after SFLP for the 17 patients who provided both pre- and post-SFLP blood samples, with controls depicted as a reference. Bars represent mean ± standard deviation. Single asterisk denotes P < 0.05, double asterisk denotes P < 0.001, n.s. denotes P > 0.05. **(c)** Matched patient samples for patients with TTTS pre- and post-SFLP demonstrating changes in relative miRNA expression for each patient.

Serial assessments of miRNA expression from multiple blood draws were available for a cohort of TTTS patients (n = 17) which allowed comparison of miRNA expression before and after SFLP for treatment of TTTS (Figure 3a). All 17 patients demonstrated improvement of the TTTS physiology. Relative expression of the top 5 differentially expressed miRNAs in TTTS before and after SFLP (Figures 3b,c) demonstrated trends in expression that brought pre-SFLP values in the direction of controls for miR-26a-5p, miR-320a-3p, miR-222-3p, and miR-145-5p, but not for miR-328-3p.

### No differences in miRNA expression were present with respect to cardiomyopathy and fetal demise

To evaluate the potential of plasma miRNAs to predict TTTS disease severity, we performed subgroup analysis of TTTS patients by Quintero and Cincinnati stage, the presence of cardiomyopathy, and fetal demise, respectively. After correction, there were no differences observed in miRNA expression between those with recipient twin cardiomyopathy (n = 28) versus those without cardiomyopathy (n = 4) (Supplementary Table S2). Furthermore, no differences in miRNA expression were observed when comparing TTTS patients with donor or recipient demise (n = 15) to patients with dual survivorship (n = 22) (Supplementary Table S3). Finally, no differences were observed comparing Quintero stages or Cincinnati stage (all P > 0.05).

### Gene set enrichment analysis reveals putative pathways regulated by candidate miRNAs

**FIGURE 4:**
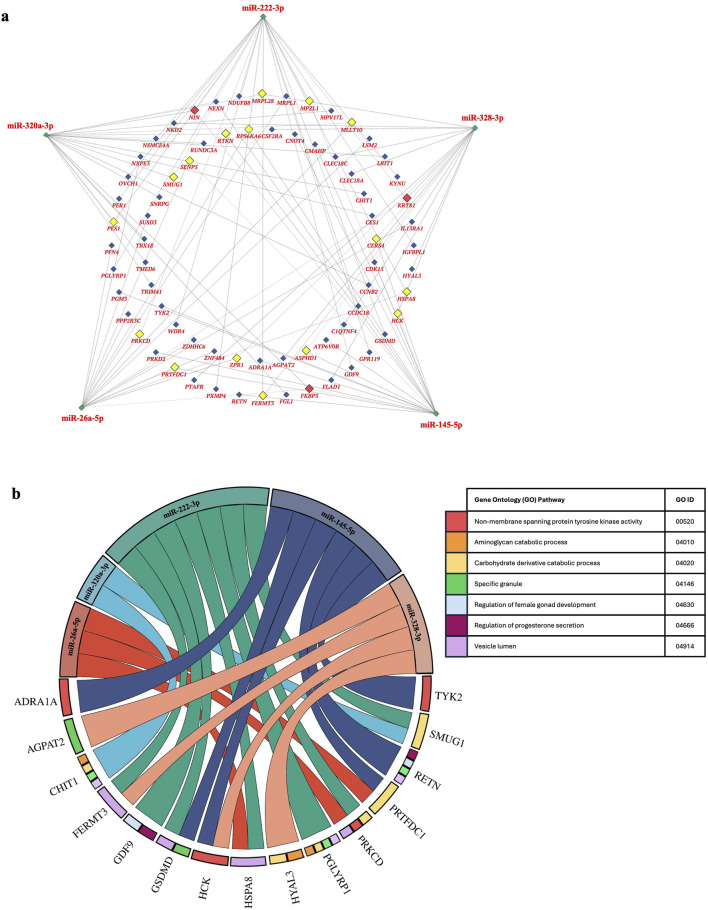
Network of genes regulated by the top 5 differentially expressed miRNAs in TTTS and putative gene ontogeny pathways. **(a)**. Network analysis depicts the top 5 differentially expressed miRNAs in TTTS (green) and respective genes regulated by three (red), two (yellow), or one (blue) miRNAs. **(b)**. Chord diagram depicting gene ontogeny (GO) pathways enriched in gene:miRNA sets.

Gene-miRNA network analysis was performed to assess the relationship between the top 5 differentially expressed miRNAs in TTTS. 71 unique genes were identified as putative targets of these miRNAs, with 18 genes regulated by two miRNAs and three genes (FKBP3, KRT81 NIN) regulated by three miRNAs, respectively (Figure 4a). Gene set enrichment analysis of the above target genes demonstrated overrepresentation in pathways including progesterone secretion, female gonad development, protein tyrosine kinase activity, specific granule and vesicle lumen activity, aminoglycan and carbohydrate catabolism (Figure 4b).

## Discussion

In this study, we performed an exploration of potential non-invasive biomarkers for twin-twin transfusion syndrome, given the heterogeneity of ultrasound-based staging syndromes for prognostication. The findings of this study suggest that differential expression of circulating miRNA is present in the maternal plasma of patients with TTTS compared to uncomplicated MCDA twin pregnancies, and furthermore, that specific miRNAs in combination demonstrate strong predictive capacity to distinguish TTTS from uncomplicated MCDA twin pregnancies. Compared to amniotic fluid, the identification of circulating biomarkers for TTTS has been extremely limited, with prior studies only showing differences in circulating angiogenic factors in TTTS patients (Fox et al., 2010; Yinon et al., 2014). A prior discovery study examining circulating miRNA profiles in TTTS did not identify any differences (Mackie et al., 2019). However, the precedent for circulating miRNAs to serve as biomarkers for TTTS has remained given their established relevance for other diseases of the fetus and placenta, suggesting the potential for transplacental transfer of miRNAs into maternal circulation (Higashijima et al., 2013; Mackie et al., 2019; Subramanian et al., 2023; Gu et al., 2019). In the present study, we identified four miRNAs, miR-26a-5p, miR-320a-3p, miR-222-3p, and miR-145-5p, that are differentially expressed in patients with TTTS compared to MCDA controls.

The identification of circulating miRNAs capable of differentiating TTTS from controls is significant and reveals a potential biomarker source with translational relevance. In addition, we found the top differentially expressed miRNAs, miR-26a-5p, miR-320a-3p, and miR-222-3p, are highly predictive of the presence of TTTS in combination. While the clinical utility of biomarkers in TTTS would be enhanced by their ability to discriminate based on disease severity, we did not find differences in expression according to several metrics of severity: cardiomyopathy, fetal demise, Quintero stage or Cincinnati stage. However, it is likely that this study was underpowered to detect true differences between subgroups as smaller patient subsets were assessed. An interesting future study would be evaluation of plasma miRNA expression in those patients with initially normal or equivocal ultrasound-based signs who progressed to TTTS on subsequent ultrasounds. First-trimester ultrasound screening to predict future development of TTTS utilizing features such as fetal nuchal translucency thickness and crown rump length discordance had significant false positive and false negative rates in prior studies (Mackie et al., 2017; Kagan et al., 2007; El Kateb et al., 2007; Sebire et al., 2000). Therefore, these patients could benefit from the identification of biochemical differences early in gestation that may predict TTTS development, aiding in risk stratification and/or earlier treatment. Importantly, we evaluated whether miRNA expression changes in response to treatment, and in paired patient samples of maternal plasma prior to and after SFLP, the expression of miR-26a-5p, miR-320a-3p, miR-222-3p and miR-145-5p corrected to levels closer to that of controls after treatment. Though outside of the scope of the present study, it is of considerable interest to assess whether plasma miRNA normalization after SFLP may predict post-procedure prognosis, as this study suggests that treatment of TTTS physiology may induce changes at the level of miRNA expression.

The miRNAs included in the panel were selected due to their relevance in other fetal and placental disease states. All four miRNAs that were significantly dysregulated are known to be dysregulated in other gestational diseases including preeclampsia; specifically, miR-320a-3p and miR-222-3p are dysregulated in trophoblast cells of patients with preeclampsia (Han et al., 2017; Xie et al., 2019; Yang et al., 2022). miR-26a-5p, which regulates uterine epithelial remodeling and immune signaling during implantation, is also dysregulated in pregnancy complications such as placenta previa and IUGR, and modulates inflammation and oxidative stress via NF-κB and cytokine pathways in other disease contexts, supporting its potential role as a pregnancy-relevant immune regulator (Hromadnikova et al., 2015; Myszczynski et al., 2025; Hromadnikova et al., 2024; Bian et al., 2024). The source of the circulating miRNAs is not known, and the differential expression could represent changes at the fetal, placental, or maternal level given that bidirectional miRNA trafficking occurs at the maternal-fetal interface (Xu et al., 2021; Morales-Prieto et al., 2020; Chang et al., 2017). In this study, we aimed to understand the potential interrelatedness of these differentially expressed miRNAs that could guide further research of downstream mediators of disease. To this aim, gene-miRNA network analysis revealed 18 genes that were simultaneously regulated by two miRNAs and three genes regulated by three miRNAs. Each of these three genes, KRT8, NIN, and FKBP3 are expressed in the human placenta, though their potential relevance to TTTS pathophysiology has not yet been elucidated (Gerli et al., 2024). Interestingly, a meta-analysis of single-cell RNA sequencing studies within placental samples identified KRT8 as the sole gene that is universally expressed within all trophoblast cell subtypes (Derisoud et al., 2024). Furthermore, KRT8 knockout mouse models produced embryonic lethality due to impairment in trophoblast giant cell layer formation (Jaquemar et al., 2003). The present study raises new questions of whether differences in miRNA expression could affect regulation of structural genes such as KRT8 implicated in placental development, an interesting avenue for further study. Additionally, it raises the question of whether in utero miRNA delivery could be a potential future treatment strategy aimed at placental modulation, as has been demonstrated in other fetal pathologies such as congenital diaphragmatic hernia (Ullrich et al., 2023; Khoshgoo et al., 2019). Interestingly, gene set enrichment analysis revealed the overexpression of target genes within several pathways of established and potential significance in TTTS pathophysiology including progesterone regulation, carbohydrate catabolism, and non-membrane spanning protein tyrosine kinase activity (Dunn et al., 2016; Hoffman et al., 2020; Parchem et al., 2023; Kajiwara et al., 2022). The mechanisms governing differences in steroid hormone expression and carbohydrate metabolism in TTTS are incompletely understood, and it is interesting to speculate whether miRNAs could be implicated in regulation of these critical fetal and placental developmental pathways (Dunn et al., 2016; Hoffman et al., 2020).

The strengths of this study include the use of a non-invasive biomarker source as most biomarker research for TTTS has been performed in amniotic fluid samples. Further strengths were the selection of uncomplicated MCDA twins as controls, multiple testing correction, and collection of samples before and after SFLP. However, this study has several limitations. First, though this study aimed to understand whether miRNAs could predict the presence of TTTS via random forest modeling, the study was retrospective and limited by sample size. Therefore, conclusions cannot be drawn on whether these miRNAs could prospectively predict disease development. Further, it is not known whether the changes in miRNA expression are unique to TTTS or could be present in other gestational twin pathologies. Several miRNAs of potential relevance to TTTS were not included in the panel due to limitations of the size of custom microarray cards, and therefore this study is not a comprehensive evaluation of all miRNAs that may be differentially expressed in maternal plasma. Assessment of a more comprehensive miRNA profile with additional validation experiments in well-powered patient cohorts is a goal of future study. Additionally, lack of an endogenous control is a limitation of the study as the intended endogenous control was not present in the samples. Although endogenous controls have not been established for this population, we hypothesize that this is due to a technical error in manufacturing of the custom plate. Finally, this study was limited in its ability to compare miRNA expression according to TTTS stage. The ability of maternal plasma miRNAs to complement existing staging systems requires prospective investigation.

We conclude that differential expression of miRNAs is present in maternal plasma of patients with TTTS compared to MCDA controls. miR-26a-5p, miR-320a-3p, miR-222-3p and miR-145-5p are significantly dysregulated in patients with TTTS. miR-26a-5p, miR-320a-3p, and miR-222-3p demonstrate strong predictive capacity for TTTS in a random forest model.

## Data Availability

The original contributions presented in the study are included in the article/Supplementary Material, further inquiries can be directed to the corresponding author.
