# Supplementary material for: Maternal plasma microRNA profiles in twin-twin transfusion syndrome and normal monochorionic twin pregnancies
Source: Front Mol Biosci. 2025 Jul 23;12:1597215. doi: 10.3389/fmolb.2025.1597215 (PMC12325072; doi:10.3389/fmolb.2025.1597215)
Supplement: Supplementary file 1 [file Supplementaryfile1.docx]

**Supplementary Table S1. Clinical characteristics of control and TTTS pregnancies**

| **Subject Number** | **GA at Initial Ultrasound (weeks)** | **GA at SFLP (weeks)** | **Cincinnati Modification (if Stage 3)** | **Quintero Stage** | **Donor Bladder (Absent, Small, or Present)** | **Abnormal UA or DV flow**  **(No or Yes)** | **Recipient RV Tei** | **Recipient LV Tei** | **Fetal Demise (No or Yes)** |
| --- | --- | --- | --- | --- | --- | --- | --- | --- | --- |
| **Discovery Cohort** | | | | | | | | | |
| D1 | 23.43 | 24.57 | 3A | 1 | Present | No | 0.41 | 0.41 | No |
| D2 | 19.29 | 20.71 | N/A | 1 | Present | No | 0.23 | 0.40 | Yes |
| D3 | 25.71 | 25.86 | N/A | 1 | Present | No | 0.42 | 0.42 | No |
| D4 | 20.71 | 21.43 | 3A | 2 | Absent | No | 0.40 | 0.41 | Yes |
| D5 | 19.86 | 19.86 | 3A | 2 | Absent | No | 0.40 | 0.39 | No |
| D6 | 22.71 | 22.86 | 3C | 3 | Present | Yes | 0.55 | 0.54 | Yes |
| D7 | 22.00 | 22.14 | 3C | 3 | Absent | No | 0.59 | 0.58 | No |
| D8 | 22.29 | 22.29 | 3C | 3 | Small | No | 0.67 | 0.74 | Yes |
| D9 | 18.43 | 18.43 | 3C | 4 | Absent | Yes | 0.44 | 0.45 | Yes |
| D10 | 20.57 | 20.57 | 3C | 2 | Absent | No | 1.59 | 0.39 | No |
| D11 | 24.00 | 25.14 | 3B | 2 | Present | No | 0.59 | 0.61 | No |
| **Normal Monochorionic Twin Control Subjects** | | | | | | | | | |
| C1 | 15.00 | N/A | N/A | N/A | Present | No | 0.34 | 0.33 | Yes |
| C2 | 17.43 | N/A | N/A | N/A | Present | No | 0.25 | 0.23 | No |
| C3 | 23.57 | N/A | N/A | N/A | Present | No | 0.37 | 0.39 | No |
| C4 | 20.29 | N/A | N/A | N/A | Present | No | 0.43 | 0.41 | No |
| C5 | 23.86 | N/A | N/A | N/A | Present | No | 0.41 | 0.40 | No |
| C6 | 16.29 | N/A | N/A | N/A | Present | Yes | 0.24 | 0.35 | No |
| C7 | 22.29 | N/A | N/A | N/A | Present | No | 0.26 | 0.29 | No |
| C8 | 17.57 | N/A | N/A | N/A | Present | No | 0.23 | 0.41 | No |
| C9 | 20.86 | N/A | N/A | N/A | Present | No | 0.33 | 0.37 | No |
| C10 | 22.43 | N/A | N/A | N/A | Present | No | 0.36 | 0.42 | No |
| C11 | 17.71 | N/A | N/A | N/A | Present | No | 0.38 | 0.41 | No |
| **TTTS Cohort** | | | | | | | | | |
| T1 | 22.14 | 22.14 | 3C | 3 | Absent | Yes | Unavailable | 0.64 | No |
| T2 | 16.57 | 16.71 | 3C | 3 | Absent | No | 0.65 | 0.59 | No |
| T3 | 22.71 | 22.86 | 3C | 3 | Present | Yes | 0.55 | 0.54 | Yes |
| T4 | 25.43 | 25.57 | 3C | 2 | Absent | Yes | 0.70 | 0.73 | Yes |
| T5 | 20.71 | 21.43 | 3A | 2 | Absent | No | 0.40 | 0.41 | Yes |
| T6 | 17.14 | 18.29 | 3C | 3 | Absent | Yes | 0.66 | 0.59 | Yes |
| T7 | 20.00 | 26.57 | 3B | 1 | Present | No | 0.44 | 0.35 | No |
| T8 | 25.14 | 25.43 | 3C | 1 | Present | No | 0.59 | 0.61 | No |
| T9 | 18.29 | 18.86 | 3B | 3 | Small | No | 0.57 | 0.37 | Yes |
| T10 | 18.57 | 18.57 | 3A | 3 | Small | Yes | 0.35 | 0.30 | Yes |
| T11 | 22.29 | 22.43 | N/A | 2 | Absent | No | 0.44 | 0.43 | No |
| T12 | 17.14 | 17.29 | 3A | 3 | Absent | Yes | 0.53 | 0.43 | Yes |
| T13 | 22.29 | 22.29 | 3C | 3 | Small | No | 0.67 | 0.74 | Yes |
| T14 | 16.71 | 17.43 | 3B | 3 | Absent | Yes | 0.49 | 0.51 | No |
| T15 | 19.86 | 19.86 | 3A | 2 | Absent | No | 0.40 | 0.39 | No |
| T16 | 18.14 | 18.29 | 3C | 1 | Small | No | 0.67 | 0.48 | No |
| T17 | 19.14 | 20.29 | 3B | 3 | Small | No | 0.52 | 0.48 | Yes |
| T18 | 22.43 | 22.57 | N/A | 3 | Absent | Yes | Unavailable | Unavailable | No |
| T19 | 21.00 | 21.14 | 3B | 3 | Small | No | 0.57 | 0.52 | No |
| T20 | 24.71 | 25.00 | 3B | 1 | Small | No | 0.55 | 0.50 | No |
| T21 | 20.57 | 20.57 | 3C | 2 | Absent | No | 1.59 | 0.39 | No |
| T22 | 22.71 | 22.71 | N/A | 3 | Absent | Yes | Unavailable | Unavailable | Yes |
| T23 | 22.57 | 22.71 | 3B | 1 | Present | No | 0.37 | 0.52 | No |
| T24 | 19.29 | 20.71 | N/A | 1 | Present | No | 0.23 | 0.40 | Yes |
| T25 | 21.29 | 21.43 | 3B | 1 | Present | No | 0.50 | 0.48 | No |
| T26 | 23.43 | 24.57 | 3A | 1 | Present | No | 0.41 | 0.41 | No |
| T27 | 16.71 | 17.00 | 3A | 3 | Absent | Yes | 0.50 | 0.41 | No |
| T28 | 19.57 | 19.57 | 3A | 3 | Absent | Yes | 0.32 | 0.35 | Yes |
| T29 | 25.71 | 25.86 | N/A | 1 | Present | No | 0.42 | 0.42 | No |
| T30 | 22.57 | 22.57 | 3C | 3 | Small | No | 0.52 | 0.54 | Yes |
| T31 | 16.43 | 18.00 | 3B | 3 | Small | Yes | 0.44 | 0.50 | No |
| T32 | 22.14 | 23.57 | N/A | 3 | Absent | Yes | Unavailable | Unavailable | Yes |
| T33 | 16.86 | 17.86 | 3C | 2 | Small | Yes | 0.58 | 0.60 | No |
| T34 | 18.43 | 19.29 | N/A | 2 | Absent | No | 0.44 | 0.41 | No |
| T35 | 16.43 | 18.71 | 3B | 3 | Absent | No | 0.51 | 0.49 | Yes |
| T36 | 18.00 | 20.00 | 3A | 3 | Small | Yes | 0.41 | 0.45 | No |

**Supplementary Table S2:** Differential expression of top 5 miRNAs in patients with cardiomyopathy vs. no cardiomyopathy

| **Assay** | **Mean ∆CT Cardio (SD)** | **Mean ∆CT No Cardio (SD)** | **∆∆CT**  **(Cardio – No Cardio)** | **Fold Change**  **(Cardio/No Cardio)** | ***P-*value** | **B-H Adj.**  ***P*-value** |
| --- | --- | --- | --- | --- | --- | --- |
| hsa-miR-145 | -0.59 (1.09) | -1.90 (0.95) | 1.31 | 0.40 | *0.040* | 0.420 |
| hsa-miR-24 | 0.22 (0.61) | 0.88 (0.47) | -0.66 | 1.58 | *0.040* | 0.420 |
| hsa-miR-let7b | 0.57 (0.86) | -0.22 (0.58) | 0.79 | 0.57 | 0.072 | 0.504 |
| hsa-miR-92a | 0.23 (1.60) | -0.77 (0.95) | 1.00 | 0.50 | 0.135 | 0.592 |
| hsa-miR-146a | 0.30 (0.58) | -0.80 (0.40) | 1.10 | 0.47 | 0.169 | 0.592 |

**Supplementary Table S3:** Differential expression of top 5 miRNAs in patients with fetal mortality versus no fetal mortality

| **Assay** | **Mean ∆CT Mortality (SD)** | **Mean ∆CT No Mortality (SD)** | **∆∆CT**  **(Mortality – No Mortality)** | **Fold Change**  **(Mortality/No Mortality)** | ***P-*value** | **B-H Adj.**  ***P*-value** |
| --- | --- | --- | --- | --- | --- | --- |
| hsa-miR-let7b | -0.80 (0.75) | -0.36 (0.95) | -0.45 | 1.25 | 0.117 | 0.714 |
| hsa-miR-222 | -.58 (0.40) | -0.81 (0.48) | 0.22 | 0.84 | 0.141 | 0.714 |
| hsa-miR-24 | 0.98 (0.61) | 0.63 (0.68) | 0.35 | 0.76 | 0.160 | 0.714 |
| hsa-miR-16 | -2.30 (0.95) | -1.78 (1.01) | -0.52 | 1.38 | 0.170 | 0.714 |
| hsa-miR-146a | 0.24 (0.66) | -0.05 (0.60) | 0.29 | 0.82 | 0.238 | 0.714 |
