# Supplementary figures and images for: Maternal plasma microRNA profiles in twin-twin transfusion syndrome and normal monochorionic twin pregnancies
Source: Front Mol Biosci. 2025 Jul 23;12:1597215. doi: 10.3389/fmolb.2025.1597215 (PMC12325072; doi:10.3389/fmolb.2025.1597215)

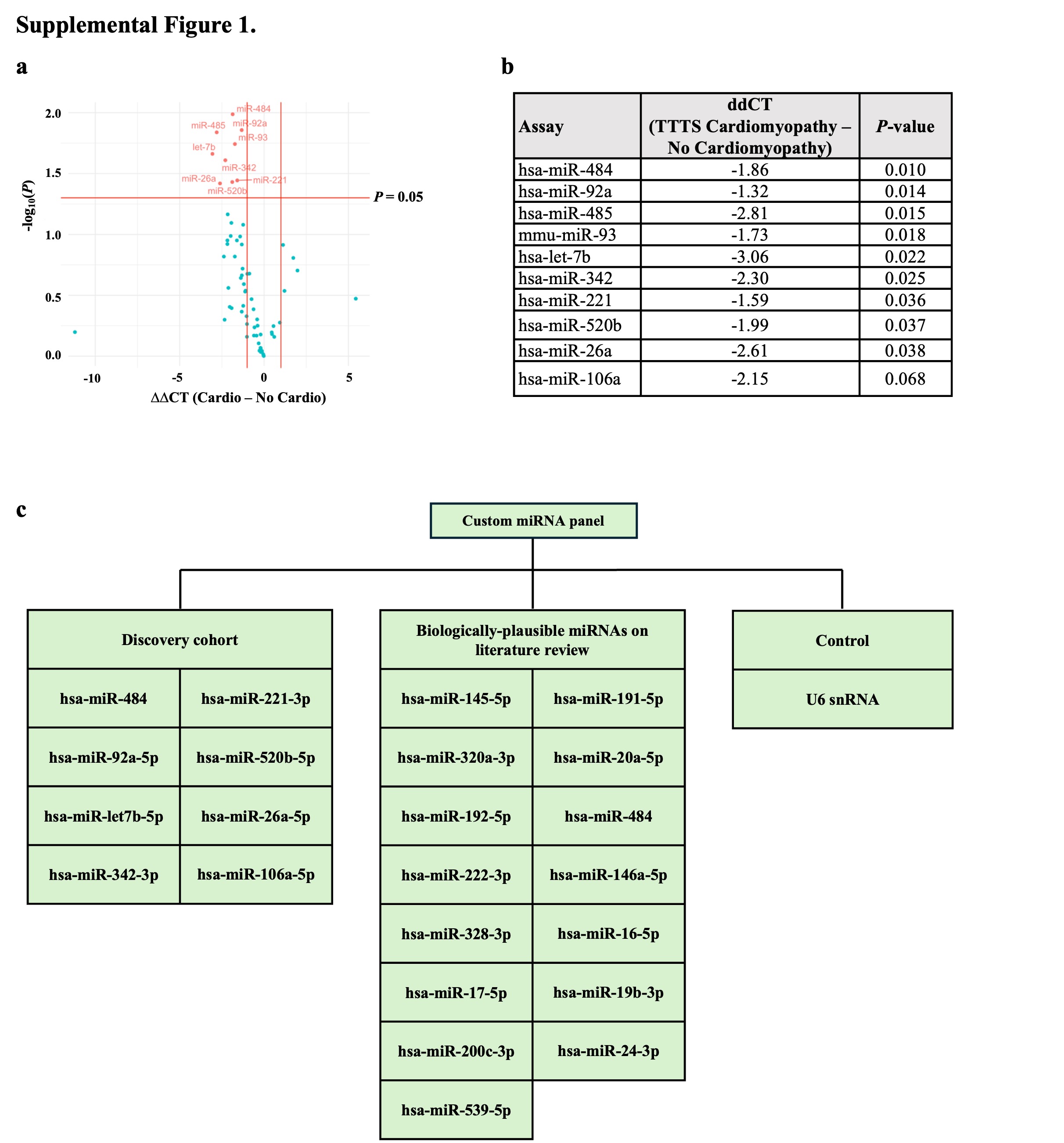

Supplement: Supplementary file 2 [file Image1.jpeg]
